# Myeloperoxidase Oxidized LDL Interferes with Endothelial Cell Motility through miR-22 and Heme Oxygenase 1 Induction: Possible Involvement in Reendothelialization of Vascular Injuries

**DOI:** 10.1155/2014/134635

**Published:** 2014-11-02

**Authors:** Jalil Daher, Maud Martin, Alexandre Rousseau, Vincent Nuyens, Hussein Fayyad-Kazan, Pierre Van Antwerpen, Guy Courbebaisse, Philippe Martiat, Bassam Badran, Frank Dequiedt, Karim Zouaoui Boudjeltia, Luc Vanhamme

**Affiliations:** ^1^Institute for Molecular Biology and Medicine (IBMM), Université Libre de Bruxelles, rue des Professeurs Jeener et Brachet 12, 6041 Gosselies, Belgium; ^2^Laboratory of Protein Signaling and Interactions, Interdisciplinary Cluster for Applied Genoproteomics (GIGA-R), University of Liège, Avnue de l'Hopital 1 (B34), 4000 Sart-Tilman, Belgium; ^3^Laboratory of Experimental Medicine (ULB 222 Unit), CHU de Charleroi, A. Vésale Hospital, Université Libre de Bruxelles, rue de Gozée 706, 6110 Montigny-le-Tilleul, Belgium; ^4^Laboratory of Experimental Hematology, Institut Jules Bordet, Université Libre de Bruxelles, Boulevard de Waterloo 121, 1000 Bruxelles, Belgium; ^5^Laboratory of Pharmaceutical Chemistry, Faculty of Pharmacy, Université Libre de Bruxelles, Boulevard du Triomphe, Campus Plaine CP 205/5, 1050 Bruxelles, Belgium; ^6^Creatis, CNRS UMR 5220, INSERM U1044, UCB Lyon1, INSA Lyon, University of Lyon, 7 Avenue Jean Capelle, 69621 Villeurbanne, France; ^7^Laboratory of Immunology, Department of Biochemistry, Faculty of Sciences, Lebanese University, Hadath, Beirut 21219, Lebanon

## Abstract

Cardiovascular disease linked to atherosclerosis is the leading cause of death worldwide. Atherosclerosis is mainly linked to dysfunction in vascular endothelial cells and subendothelial accumulation of oxidized forms of LDL. In the present study, we investigated the role of myeloperoxidase oxidized LDL (Mox-LDL) in endothelial cell dysfunction. We studied the effect of proinflammatory Mox-LDL treatment on endothelial cell motility, a parameter essential for normal vascular processes such as angiogenesis and blood vessel repair. This is particularly important in the context of an atheroma plaque, where vascular wall integrity is affected and interference with its repair could contribute to progression of the disease. We investigated *in vitro* the effect of Mox-LDL on endothelial cells angiogenic properties and we also studied the signalling pathways that could be affected by analysing Mox-LDL effect on the expression of angiogenesis-related genes. We report that Mox-LDL inhibits endothelial cell motility and tubulogenesis through an increase in miR-22 and heme oxygenase 1 expression. Our *in vitro* data indicate that Mox-LDL interferes with parameters associated with angiogenesis. They suggest that high LDL levels in patients would impair their endothelial cell capacity to cope with a damaged endothelium contributing negatively to the progression of the atheroma plaque.

## 1. Introduction

Cardiovascular disease (CVD) is the leading cause of death worldwide. According to the 2005 World Health Organization report, CVD is responsible for 30% of all deaths globally. CVD commonly stems from vascular dysfunction, which is mainly linked to atherosclerosis. The accumulation of oxidized forms of LDL (ox-LDL) in the intima of blood vessels is widely accepted to play a major role in the development of atheroma plaques. LDL particles are oxidized* in situ*; small amounts can also be found in the circulation and are therefore in contact with the endothelial cells (ECs) [1–3]. Wherever these particles are modified, myeloperoxidase (MPO) is nowadays the best candidate as an* in vivo* actor in LDL modification whereas other candidates (such as lipoxygenases, NADPH oxidases, and endogenous copper) were previously considered [4]. MPO is an enzyme secreted by activated neutrophils; it catalyses the production of hypochlorous acid from hydrogen peroxide and chloride. The generated HOCl is responsible for the modification of LDLs into chlorinated LDLs (HOCl-LDLs) [5].

Furthermore, serum MPO levels are markers of adverse prognosis in patients with acute coronary syndromes [6]. Mox-LDLs are endowed with proinflammatory properties. Mox-LDL indeed triggers IL-8 gene expression by endothelial cells and monocytes and macrophages, respectively [4]. They also interfere with EC function. The endothelial dysfunction involves expression of adhesion molecules recruiting immune cells to the endothelium and allowing them to penetrate into lesions [7]. Among the recruited cell types are monocytes, which, once in the intima, will differentiate into macrophages, engulf atherogenic oxidized lipoproteins, and evolve into the so-called foam cells. Foam cell accumulation contributes to the formation of an atheroma core which will further evolve into a mass of necrotic cells, cholesterol, and lipid debris [8]. This process is considered the hallmark of early atherosclerosis. Endothelial dysfunction also impedes EC motility and migration [9, 10]; EC motility is a fundamental aspect of angiogenesis, an essential process involved in many physiological and pathological processes [11–13]. It could also be critical in cases of endothelium desquamation associated with the presence of circulating ECs correlated to many pathologies such as vasculitis, lupus erythematosus, sickle cell anemia, thalassemia, septic shock, infections (rickettsiae, CMV), or cancer [14]. As far as atherosclerosis is concerned, circulating ECs are associated with plaque ruptures, stroke, or myocardial infarctions [15]. Physical denudation of the ECs bed has been described at atheroma plaque locations and a failure to locally regenerate the endothelium is suspected to be deleterious to the endothelium function [16]. Also post-AMI (acute myocardial infarction) vascular reconstruction surgery and stenting procedures have been doomed to failure due to restenosis which is thought to be linked to the inability of the endothelium to regenerate and heal the denuded regions [13]. Another phenomenon involving EC growth and associated with CVD is stent reendothelialization. Finally, most of the procedures involved in stenosis or aneurysm treatments involve catheterization associated with vascular wall denudation [17].

The question arises whether oxidized LDL, on top of being instrumental in atheroma plaque formation, would also interfere with the ability to heal the associated endothelium whether this damage is subsequent to atherosclerosis or not. In order to address this question, we investigated the possible effect of physiologically modified LDL on EC motility and survival as well as gene expression and signal transduction. We report that pathological concentrations of MPO-modified LDLs inhibit EC mobility and angiogenic properties and that this defect relies on miR-22 and heme oxygenase 1 (HO-1) increase.

## 2. Material and Methods

### 2.1. Cell Culture, Transfection, and Gene Knockdown and Overexpression

Clonetics HUVEC Cells (Lonza) were cultured in complete Clonetics EBM Endothelial Growth Medium (Lonza; Cat. number CC-312) at 37°C, 5% (v/v) CO_2_ according to the provider's recommendations. Cell transfections were carried out using Gene Trans II according to the manufacturer's instructions (MobiTec; Cat. number 0102B). For miR-22 selective knockdown and inhibition, we used miRCURY LNA microRNA inhibitor [hsa-miR-22 ^*^] supplied by Exiqon (product number 411669-00) (sequence: AAGCTTGCCACTGAAGAAC). For quality assessment of the knockdown experiment, we used a miRCURY LNA microRNA inhibitor negative control (sequence: GTGTAACACGTCTATACGCCCA). For HO-1 overexpression, we used the TrueClone pCMV6AC-HMOX1(NM_002133.1) vector supplied by OriGene (SKU SC320297).

### 2.2. Measuring Cell Behavior Using the xCELLigence System

The detailed protocol was thoroughly described previously [18]. Briefly, 50 *μ*L of EBM at room temperature was added into each well of E-plate 16. The E-plate 16 was then connected to the system and checked in the cell culture incubator for proper electrical contacts and the background impedance was measured during 24 s. HUVECs were resuspended in EBM and adjusted to 10^3^ cells/*µ*L. 100 *μ*L of the cell suspension was added to the wells on E-plate 16 and the plate was then placed back to the incubator. Approximately 24 hours after seeding, the cells were exposed to 100 *μ*L of control medium or medium containing either native LDL or Mox-LDL at a final concentration of 100 *μ*g/mL. Controls received EBM medium only. Cell behavior was monitored every 20 min by the xCELLigence system for a period of 48 hours via the incorporated sensor electrode arrays of the E-Plate 16. The electrical impedance was measured by the RTCA-integrated software of the xCELLigence system as a dimensionless parameter termed Cell Index (CI).

### 2.3. Tubulogenesis Assay

The Matrigel tube formation assay, or tubulogenesis assay, was performed to assess the ability of HUVECs to form endothelial cell vascular structures (or tubules) involved in vessel formation (angiogenesis). As previously described [19], individual wells were coated with 100 *μ*L of Matrigel Basement Membrane Matrix (BD Biosciences; Cat. number 356234) in a 24-well plate and allowed to polymerize at 37°C for 2 hours. After polymerization, 250 *μ*L of complete EBM was added to each well and the plate was incubated for an additional hour at 37°C. 5 × 10^4^ HUVECs (which were either treated or not with native LDL and Mox-LDL for 24 hours) were then resuspended in 500 *μ*L of complete EBM (Lonza; Cat. number CC-312) (± native LDL or Mox-LDL at a final concentration of 100 *µ*g/mL) and were added to each well in duplicate. After an overnight incubation, cells were inspected for tubule formation and pictures were taken from three fields of views using inversed light microscopy with the 4x objective. The tubule networks lengths were measured using the Image J program, averaged between the fields of views and duplicates, and expressed as a percentage of control untreated cells. Experiments were reproduced 3 times independently.

### 2.4. Tubulogenesis in Real-Time

The procedure was conducted as described above with the exception that the cells were incubated in a 37°C conditioned chamber of an AxioVision-Carl Zeiss microscope and the microscope was set to automatically take pictures at 10 min intervals over a 9-hour period. The culture medium was buffered with HEPES at a 15 mM final concentration to compensate for the CO_2_ unavailability in our setup.

### 2.5. *In Vitro* Wound-Healing Assay

HUVEC migration was monitored using the wound-healing assay as previously described [20]. Briefly, 7 × 10^4^ cells/well/mL were seeded in 24-well plates in EBM medium (± native LDL or Mox-LDL at a final concentration of 100 *µ*g/mL). After a 24-hour incubation allowing the cells to attach and form a confluent monolayer, the cells were scraped with a conserved width using a yellow tip. After scraping, cells were washed with PBS and were incubated in EBM with or without native LDL/Mox-LDL at final concentrations of 100 *µ*g/mL. Scratch width was photographed at times 0 and 8 hours from three fields of views using the 20x objective with a microscopic camera system coupled to an inverted light microscope. The experiments were performed in duplicate. Using the Image J program, the wound sizes were measured by calculating the distance between the wound edges which remained cell-free at *t* = 8 h, averaged between the fields of views and duplicates and expressed as a percentage of the initial wound width at *t* = 0 h.

### 2.6. Cell Migration Assay

As previously described [21], cell migration assays were carried out using modified Boyden chambers consisting of Transwell membrane filter inserts (Greiner Bio-one; Cat. number 662631) in 24-well tissue culture plates. The translucent Transwell membranes were 6.5 mm in diameter with a pore size of 8 *μ*m. The upper surfaces of the Transwell membranes were coated with fibronectin and the Transwells were initially incubated for 2 hours at 37°C and then overnight at 4°C. The second day, the inserts were rinsed once with EBM medium and then placed into 24-well tissue culture plates containing 600 *μ*L of EBM medium/well. HUVECs (1.5 × 10^5^ cells treated or not treated with either native LDL or Mox-LDL for 24 hours) in 100 *μ*L of EBM medium (± native LDL or Mox-LDL at a final concentration of 100 *μ*g/mL) were seeded into the upper side of each Transwell chamber and the native LDL or Mox-LDL preparations were added simultaneously (at a final concentration of 100 *μ*g/mL) with a chemotactic agent (VEGF at 0.4 *μ*g/mL) to the lower compartment of the Boyden chambers. After incubation for 8 hr at 37°C, the cells remaining on the upper membrane surface were removed by wiping with a cotton swab, and filters were fixed with 4% paraformaldehyde and stained with crystal violet. The number of cells per membrane lower surface was counted under a light microscope using the 20x objective. The experiments were performed in duplicate and the numbers of invaded cells were averaged between the duplicates and expressed as a fold/control untreated cells.

### 2.7. Study of Cell Death and Cell Proliferation by Flow Cytometry

Confluent HUVECs were either treated or not treated with native LDL or Mox-LDL at a final concentration of 100 *μ*g/mL for 24 hours. Cell death was detected using the LIVE/DEAD Fixable Red Dead Cell Stain Kit (Molecular Probes; Cat. number L-23102) following manufacturer's instructions. HUVECs were harvested and stained with the near-IR Dead Cell Stain which is excited at a 633/635 nm wavelengths. For proliferation analysis, BD Pharmingen Ki67 kit was used and the assay was also performed according to manufacturer's instructions. In brief, after staining with the Red Dead Cell Stain, cells were fixed, permeabilized, and then stained with FITC-Ki67 (BD Pharmingen, San Diego, CA, USA; Cat. number 612472) for 20 min at 4°C. Cells were then analyzed using BD FACSCanto II flow cytometry, and data were analyzed using FlowJO (Tree Star, inc.©). The experiments were performed in duplicate and the percentages of proliferating and dead cells were averaged between the duplicates.

### 2.8. PI/Annexin V Staining and Flow Cytometry Analysis to Study Apoptosis

HUVECs were either treated or not treated with native LDL or Mox-LDL at a final concentration of 100 *μ*g/mL for 24 hours. Apoptosis was detected using BD Pharmingen FITC Annexin V Detection Kit 1. The assay was performed according to manufacturer's instructions. In brief, HUVECs were washed twice with cold PBS and resuspended in Binding Buffer at a concentration of 10^6^ cells/mL. FITC Annexin V and PI were added and cells were vortexed gently and incubated in the dark at room temperature for 15 min. Flow cytometry analysis was performed within 1 hour using BD FACSCanto II flow cytometry, and data were analyzed using FlowJO (Tree Star, inc.©). The experiments were performed in duplicate and the percentages of annexin V positive and PI positive cells were averaged between the duplicates.

### 2.9. Western Blot Analysis of Phospho-FAK

HUVECs were either treated with mock medium or Mox-LDL at a final concentration of 100 *μ*g/mL for 24 hours. Cells were then solubilized in Laemmli Sample Buffer. Total cellular protein extracts were separated by SDS-PAGE, electrotransferred to PDVF membranes, analyzed with anti-p-FAK (Tyr 576)-R antibody (200 *µ*g/mL; 1 : 200; Santa Cruz Biotechnology), and detected with Western Lightning-ECL (PerkinElmer Inc).

### 2.10. Immunofluorescence

HUVECs were either treated with mock medium or Mox-LDL at a final concentration of 100 *μ*g/mL for 24 hours. Cells were then seeded onto fibronectin-coated coverslips. The next day, cells were fixed in 4% paraformaldehyde, permeabilized in 0.1% Triton X-100, blocked in BSA, and incubated overnight with the appropriate primary antibody. Cells were then further incubated with the corresponding secondary antibodies for 1 h. After washing, cells were mounted with Fluoro-Gel (Laborimpex) and processed for immunofluorescence using a Nikon fluorescence confocal microscope (Nikon A1R).

### 2.11. HUVECs MicroRNA Profiling and Data Analysis

HUVECs were either treated or not treated with native LDL or Mox-LDL at a final concentration of 100 *μ*g/mL for 24 hours. Total RNA was extracted from cells using TRIzol total RNA isolation reagent (Roche Applied Science). To profile microRNA expression in the treated and nontreated HUVECs, a two-step procedure was then performed. First, for cDNA synthesis from the miRNAs, 1000 ng of the extracted total RNA was subjected to reverse transcription (RT) using the TaqMan microRNA reverse transcription kit (Applied Biosystems) and Megaplex RT primers (Human Pool A, Applied Biosystems) following the manufacturer's instructions, allowing simultaneous reverse transcription of 380 mature human miRNAs. RT was performed on a Mastercycler Epgradient thermocycler (VWR). After the RT step, cDNA was diluted in RNase-free water, combined with TaqMan gene expression master mix, and loaded into the TaqMan Human MicroRNA Array A (Applied Biosystems). This microRNA array is a 384-well real-time PCR-based microfluidic card with embedded TaqMan primers and probes in each well corresponding to 380 different mature human miRNAs. Quantitative RT-PCR was performed on an ABI PRISM 7900HT sequence detection system (Applied Biosystems) according to the manufacturer's instructions. The relative expression levels of miRNAs were calculated by the SDS 2.3 software (Applied Biosystems) using the comparative ΔΔCt method and RNU48 as endogenous control.

### 2.12. TaqMan miRNA Assay for Individual miRNAs

Cells were either treated or not with native LDL or Mox-LDL at a final concentration of 100 *μ*g/mL for 24 hours and total RNA was extracted from cells using TRIzol total RNA isolation reagent (Roche Applied Science). According to the manufacturer's instructions, The TaqMan microRNA reverse transcription kit (Applied Biosystems) was used to perform gene-specific reverse transcription for each microrna using 10 ng of purified total RNA, 100 mm dNTPs, 50 units of MultiScribe reverse transcriptase, 20 units of RNase inhibitor, and 50 nm gene-specific RT primer samples. Real-time PCRs were carried out on an ABI Prism 7900HT sequence detection system (Applied Biosystems) using 5 *μ*L of the RT product, 10 *μ*L of TaqMan 2x universal PCR master mix (Applied Biosystems), 1 *μ*L of TaqMan microRNA assay mix (containing PCR primers and TaqMan probes) according to the manufacturer's instructions. All quantitative RT-PCRs were performed in triplicate and the relative expression levels of miRNAs were calculated by the SDS 2.3 software (Applied Biosystems) using the comparative ΔΔCt method.

### 2.13. Quantitative Real-Time PCR

Cells were either treated or not with native LDL or Mox-LDL at a final concentration of 100 *μ*g/mL for 24 hours. Total RNA was extracted from cells using TRIzol total RNA isolation reagent (Roche Applied Science) and reverse-transcribed using Reverse Transcriptase Core Kit (Eurogentec) according to the manufacturer's instructions. qRT-PCR was performed in 96-well plate format using SYBR Green-based detection on a Step-One-Plus machine (Applied Biosystems) with each 20 *μ*L reaction containing ~50 ng cDNA, 0.3 *μ*M sense and antisense primers, and ABsolute qPCR SYBR Green ROX Mix (Thermo Scientific). The plate was sealed and cycled under the following conditions: 95°C for 15 min, 40 cycles of 95°C for 15 s, 60°C for 30 s, and 72°C for 30 s. Each reaction was performed in triplicate and mRNA levels of RPL27 were used for normalization. The relative expression levels of mRNAs were calculated using the comparative ΔΔCt  method. PCR primers used for the quantification of HO-1 and RPL27 were as follows:

 HO-1 forward primer 5′-AAGACTGCGTTCCTGCTCAAC-3′;
 HO-1 reverse primer 5′-AAAGCCCTACAGCAACTGTCG-3′;
 RPL27 forward primer 5′-ATCGCCAAGAGATCAAAGATAA-3′;
 RPL27 reverse primer 5′-TCTGAAGACATCCTTATGACG-3′.

### 2.14. QRT-PCR Based StellARray System to Study Angiogenesis

Cells were either treated or not with native LDL or Mox-LDL at a final concentration of 100 *μ*g/mL for 24 hours. Total RNA was extracted from cells using TRIzol total RNA isolation reagent (Roche Applied Science) and reverse-transcribed using Reverse Transcriptase Core Kit (Eurogentec) according to the manufacturer's instructions. For expression analysis of 94 preselected genes involved in angiogenesis, Human Angiogenesis 96-well StellARray qPCR array (Lonza Ltd., Switzerland) was used according to manufacturer's instruction with ABsolute qPCR SYBR Green ROX Mix (Thermo Scientific) on an ABI Prism 7900HT sequence detection system (Applied Biosystems). Data were analyzed with Global Pattern Recognition Data Analysis Tool (Bar Harbor Biotechnology, Trenton, ME, USA) using the internal array control housekeeping gene expression for normalization.

### 2.15. MicroRNA Target Prediction

The microRNA:mRNA target predictions were downloaded from public websites TargetScan algorithm release 4.1 (http://www.targetscan.org) and miRanda algorithm (http://www.microrna.org).

### 2.16. Recombinant MPO Preparation

Recombinant MPO was prepared as described previously. Briefly, in order to express MPO, a recombinant plasmid that codes for prepromyeloperoxidase was constructed and named pNIV2703. This plasmid contains an MPO fragment coding for amino acid 11 in the putative signal sequence to amino acid 696. The pNIV2703 expression vector was transfected into CHO cells by electopermeabilization. Cell supernatants were recovered to assay the production level and the enzymatic activity of secreted molecules. Each batch solution was characterized by its activity (U/mL), protein concentration (mg/mL), and specific activity. Peroxidative activity was determined using *o*-dianiside as the substrate. Protein concentration was measured using the Lowry assay, with ovalbumin as a standard. Each batch was checked for endotoxin using the Lonza Endotoxin Detection Kit QCL-1000 (Catalog Number: 50-647U). Concentration was always less than 100 pg/mL, which, taking into account the final dilution of the MPO-treated LDL fraction, would contribute a final concentration of less than 0.1 pg/mL to the Mox-LDL supplemented medium added to the cells.

### 2.17. Isolation of Native LDL and Mox-LDL Preparation

Lipoprotein particles were isolated from plasma from sterile blood pouches using density-gradient ultracentrifugation. The native LDL fraction (*d* = 1.019–1.063) was stored under nitrogen at 4°C in the dark and oxidized according to the procedure described below: prior to oxidation, native LDL were gel filtered (PD-10 column, Pharmacia) and 1.6 mg of native LDL was oxidized by 2.1 chlorinating units of recombinant MPO, to generate the oxidized LDL (Mox-LDL) in the presence of 1 mM H_2_O_2_ in 2 mL PBS at pH 6.5 for 5 minutes. LDLs were desalted again after MPO treatment. Protein concentration was measured by the Lowry assay, using ovalbumin as a standard.

### 2.18. Statistical Analysis

GraphPad Prism was used for the analysis. Data were evaluated either by one-way ANOVA or Student's *t*-tests. *P* values of <0.05 (∗), <0.01 (∗∗), and <0.001 (∗∗∗) were considered significant.

## 3. Results

### 3.1. MPO-Oxidized LDL (Mox-LDL) Interferes with Cell Behavior in HUVECs

**Figure 1:**
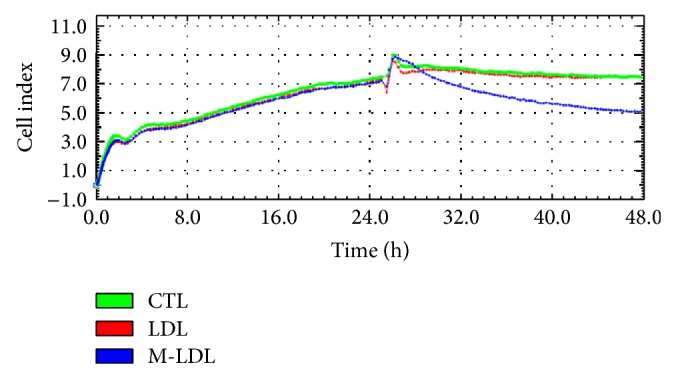
Dynamic real-time monitoring of HUVEC behavior using the xCELLigence RTCA system. Impedance Cell indexes (CIs) of HUVECs were monitored during 48 hours. Cells were seeded and incubated for 25 hours after which the medium was supplemented with native LDL, Mox-LDL, or mock medium. The result shown is representative of three independent experiments performed in duplicate.

In order to monitor in real-time the influence of Mox-LDL on the dynamics of cell adhesion and proliferation in HUVECs, the xCELLigence system [18] was exploited. Cells were seeded and the impedance of the EC layer was measured in real time. The impedance Cell Index (CI) progressively increased over 24 hours after seeding, due to intrinsic cell properties probably including a combination of cell adherence, morphological changes, and proliferation. After 24 hours of signal acquisition, cells reached confluence and covered up the whole surface area of the E plate wells and impedance reached a plateau. Native LDL or Mox-LDL was then added to the wells and the impedance measurements were carried on for another 24 hours. While the impedance CI remained stable in the mock-treated and native LDL-treated wells, it significantly decreased in the Mox-LDL treated wells (Figure 1). As the impedance CI is affected by cell adhesion, proliferation, attachment, spreading, and intercellular contact, this suggested that Mox-LDL interferes with one of these cell parameters. We therefore decided to further address these EC properties in dedicated assays.

### 3.2. Mox-LDL Decreases Tubulogenesis in HUVECs

**Figure 2:**
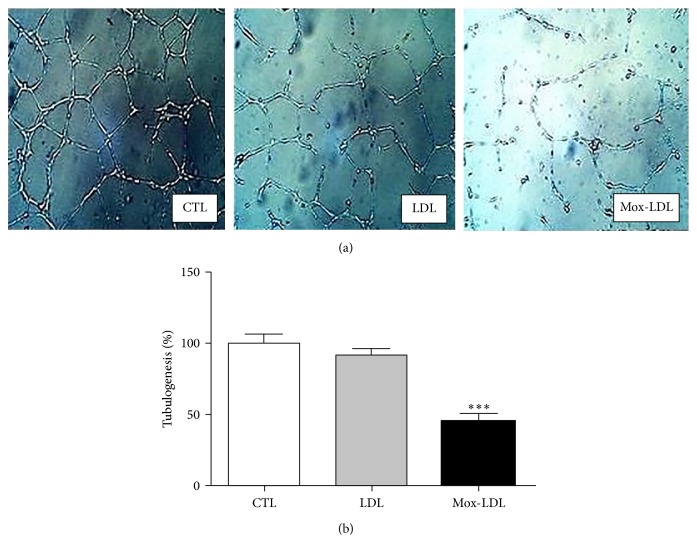
The effect of Mox-LDL treatment on HUVEC tubulogenesis. (a) Representative fields of HUVEC cultures in tubulogenic conditions. HUVECs were seeded on Matrigel supplemented with mock medium (CTL), native LDL (LDL), or Mox-LDL (MoxLDL) and incubated overnight. The fields are representative of three independent experiments performed in duplicate. (b) Quantification of tubulogenesis in HUVEC cultures. After overnight incubation in Matrigel supplemented with mock medium (CTL), native LDL (LDL), or Mox-LDL (Mox-LDL), pictures were taken of 4.906 mm square fields. Total tubule network length was then measured using the Image J computer program. Shown data are expressed as total tubule network length for each condition and normalized to the untreated control set as 100%. Mean ± SEM on 3 independent experiments performed in duplicate. ^***^ *P* < 0.001 versus control (ANOVA, Turkey's multiple comparison test).

In order to investigate the effect of Mox-LDL on the ability of ECs to build a vascular network, an* in vitro* tubulogenesis assay was performed using HUVECs. ECs were initially incubated in control medium or medium supplemented with native LDL or Mox-LDL. After 24 h, cells were switched to Matrigel coated plates overnight. As expected, control ECs formed a characteristic capillary-like network [22]. However as illustrated in Figure 2(a), Mox-LDL partially inhibited the ability of EC to build a vascular network, whereas control native LDL had no effect. Quantification analysis indicated that the decrease of the cumulative vessel length in presence of Mox-LDL was higher than 50% (Figure 2(b)), demonstrating that Mox-LDL severely affected angiogenic activities of ECs. Monitoring network formation in real time during 9 hours indicated that this effect was related to an inhibition of sprout projection rather than to regression of preformed network (data not shown).

### 3.3. Mox-LDL Inhibits Migratory Abilities of ECs* In Vitro*

**Figure 3:**
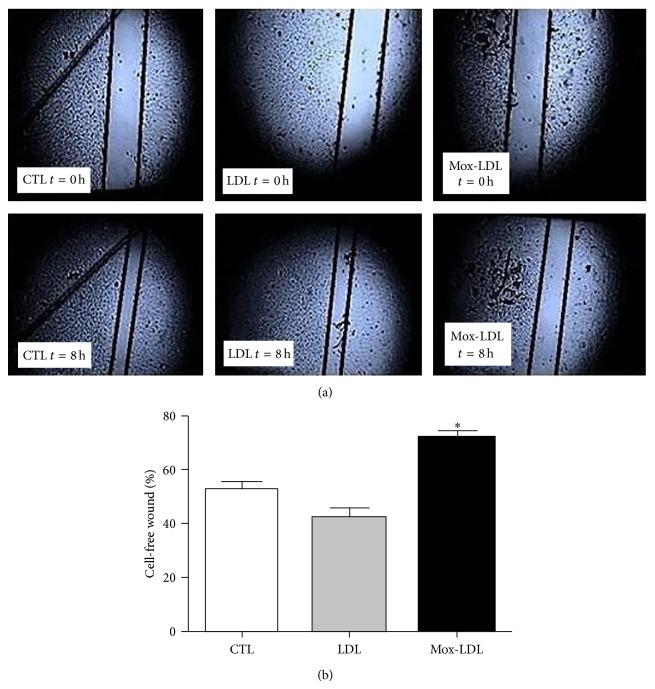
The effect of Mox-LDL treatment on wound closure after HUVEC monolayer scratch. (a) Scratched monolayers of HUVEC cells. HUVEC were seeded, allowed to reach confluence, and incubated for 24 hours more. HUVECs were then either treated or not with native LDL or Mox-LDL for 24 h. Wounds were then made by scratching the confluent cell monolayers with a yellow pipet tip and washed twice and cells were incubated for additional 8 hours in the same conditions. Pictures were taken at time of wounding and eight hours later in all conditions. Black lines defining wound edges were superimposed to the pictures. The results shown are representative of three independent experiments performed in duplicate. (b) Quantification of (a). After scratching the HUVEC monolayers to induce wounds, cells were incubated for additional 8 hours in the above conditions, pictures were taken, and the distance between the wound edges were measured in all conditions. The distance that remained cell-free at *t* = 8 h was calculated as a percentage of the initial wound width at time of scratching *t* = 0 h. Mean ± SEM on 3 independent experiments performed in duplicate. ^*^ *P* < 0.05 versus control (ANOVA, Turkey's multiple comparison test).

Because tubulogenesis is highly dependent on cell migration, we next investigated the action of Mox-LDL in an* in vitro* wound healing assay [23]. In this assay, cultured HUVEC monolayers preincubated for 24 hours in mock medium, native LDL, or Mox-LDL supplemented medium were allowed to recolonize a scratched area for 8 hours. As illustrated by Figure 3(a), the migration ability of Mox-LDL-treated ECs was decreased, an effect not seen in presence of native LDL. Cell migration was assessed by measuring the distance between the scratch edges of the cell free area at *t* = 8 h and expressing it as a percentage of the initial scratch width at *t* = 0 h. As shown in Figure 3(b), while 57% and 46% of the scratch girth remained unclosed after 8 hours in mock and LDL treated cells, respectively, 80% remained unclosed in Mox-LDL treated cells.

### 3.4. Mox-LDL Inhibits ECs Directional Chemomigration

**Figure 4:**
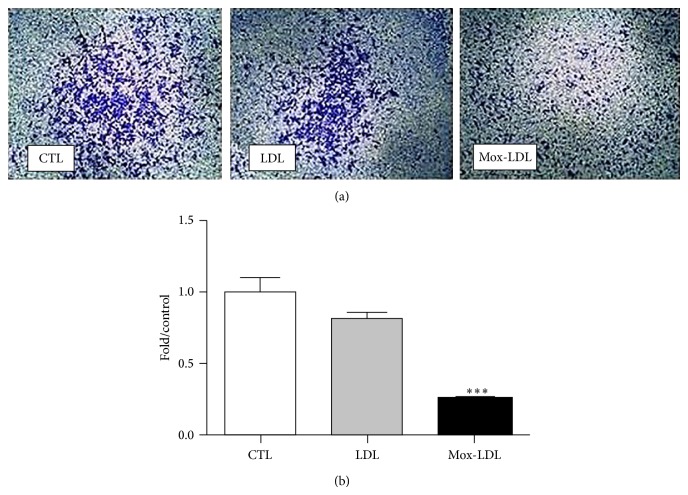
The effect of Mox-LDL treatment on HUVEC cell migration. (a) HUVEC cells which migrated through membranes. HUVEC cells were incubated for 24 hours in culture medium supplemented with mock medium, native LDL, or Mox-LDL and then seeded onto the membrane in the upper chamber of a modified Boyden chamber in conditions inducing the migration to the lower chamber. After 8 hours cells that migrated to the lower membrane side were fixed and stained. The results shown are representative of three independent experiments performed in duplicate. (b) Quantification of (a). Pictures were taken of fixed and stained cells that migrated through the membrane. Cells were counted and numbers of migrated cells were expressed as fold over control untreated cells. Mean ± SEM on 3 independent experiments performed in duplicate. ^***^ *P* < 0.001 versus control (ANOVA, Turkey's multiple comparison test).

We then evaluated the effect of Mox-LDL on HUVEC chemomigration in a modified Boyden chamber assay [24]. HUVECs were initially cultured in the presence or absence of native LDL or Mox-LDL for 24 hours. Cells were then harvested and seeded in the bottom of the upper chamber of the Boyden chamber in similar conditions. Directional cell migration was induced towards a VEGF gradient. As illustrated by Figure 4(a), EC migration was diminished in presence of Mox-LDL. In contrast, native LDL had no effect. Quantification analysis indicated that Mox-LDL treatment decreased cell migration by more than 60% (Figure 4(b)). Altogether, these results indicate that both random and directional migratory abilities of ECs were severely affected by Mox-LDL addition.

### 3.5. Mox-LDL Does Not Interfere with HUVEC Proliferation or Viability

**Figure 5:**
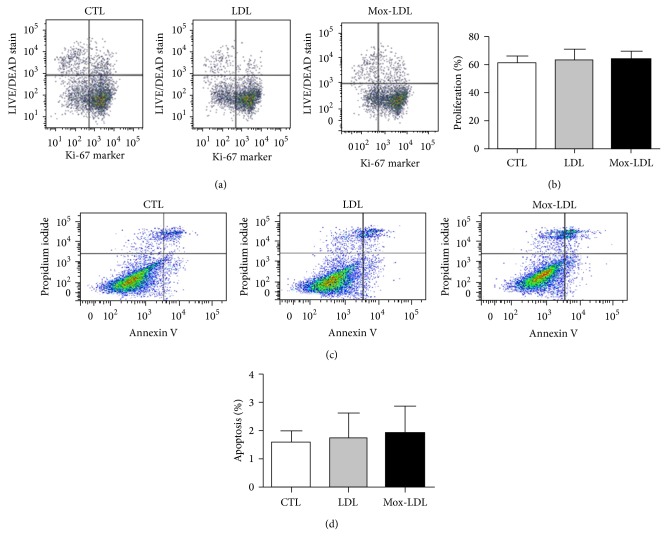
Effect of Mox-LDL treatment on HUVEC proliferation. (a) A representative Flow cytometry analysis of HUVEC cells incubated for 24 hours in culture medium supplemented with mock medium, native LDL, or Mox-LDL. Cells were then harvested and analyzed for the dead cell stain and the Ki67 proliferation marker. Percentages of cells located in the different quadrants are indicated. (b) Statistical analysis of the percentages of proliferating cells determined as in (a) on 3 independent experiments performed in duplicate. Mean ± SEM (ANOVA, Turkey's multiple comparison test). Effect of Mox-LDL treatment on HUVEC cell death. (c) A representative flow cytometry analysis of HUVEC cells incubated for 24 hours in culture medium supplemented with mock medium, native LDL, or Mox-LDL. Cells were then harvested and analyzed for PI DNA staining and phosphatidylserine annexin V staining. Percentages of cell located in the different quadrants are indicated. The results are representative of three independent experiments performed in duplicate. (d) Statistical analysis of the percentages of PI-negative annexinV positive cells determined as in (c) on 3 independent experiments performed in duplicate. Mean ± SEM (ANOVA, Turkey's multiple comparison test).

Next, we checked whether the negative effect of Mox-LDL on HUVEC tubulogenesis and migration was due to a negative effect on cell proliferation or viability. To this aim, we incubated EC in culture medium supplemented with native LDL or Mox-LDL or control medium for 24 hours. ECs were harvested and submitted to FACS analyses, first using a combination of Ki67 antibodies and LIVE/DEAD stain to evaluate cell death and proliferation [25]. The proportion of apoptotic cells was also assessed by annexin V and propidium Iodide staining [26]. No significant disparities regarding cell proliferation, viability, or death (apoptosis or necrosis) between any of the conditions (Figures 5(a) and 5(c)) could be detected. Statistical analyses of results coming from independent experiments confirmed that the percentage of proliferating, dead or PS positive cells, remained comparable in all conditions (Figures 5(b) and 5(d)).

### 3.6. Mox-LDL Has No Effect on Adhesion Foci

Our data pointed at an effect of Mox-LDL on HUVEC motility. Key players in this phenomenon are the structures mediating cells/ECM interaction called adhesion plaques or adhesion foci. We therefore analyzed the effect of our treatments on these adhesion complexes. IF staining of paxillin, a classical marker of adhesion foci, did not show any effect of Mox-LDL on the occurrence, size, or subcellular distribution of adhesion complexes in ECs as shown in Supplementary Figure I (see Supplementary Material available online at http://dx.doi.org/10.1155/2014/134635). Focal adhesion kinase (FAK) is a major player in signal transduction pathways responsible for matrix reorganization, cell-matrix adhesion, and cell migration [27]. Therefore, we investigated phosphorylation of FAK upon Mox-LDL treatment. Western blot analyses of HUVECs using an anti-phospho-FAK (Tyr 576) antibody did not reveal any effect of Mox-LDL on phosphorylated FAK levels (Supplementary Figure II) or localization (data not shown), thus confirming that Mox-LDL has no impact on EC adhesive properties.

### 3.7. Angiogenesis-Related Gene Expression Profiling in Mox-LDL Treated HUVECs

As tubulogenesis is widely accepted as an* in vitro* model of early steps of* in vivo* angiogenesis, the human angiogenesis StellARray technique was used to study the expression of 94 preselected genes involved in angiogenesis pathways. Treatment of HUVECs with Mox-LDL significantly altered the expression of four of the tested genes, including HO-1 (Supplementary Table I).

### 3.8. Transcriptional Effects of Mox-LDL Treatment in HUVECs

In order to explore the molecular mechanisms underlying Mox-LDL effects and HO-1 induction, we sought to check whether a change in microRNAs (miRs) expression patterns could be correlated with the phenotypic Mox-LDL actions in HUVECs [28]. Hence, RNA was extracted from HUVECs incubated in control or LDL and Mox-LDL supplemented medium. It was first analyzed by the TaqMan Low Density Array (TLDA) technique. Several miRs were identified to be differentially expressed exclusively in Mox-LDL treated HUVECs (see Supplementary Table II). TLDA results were validated by qRT-PCR conducted on each of the differentially expressed miRs using specific primers. This led to a high-confidence miR signature of miR-133a, miR-203, miR-22 overexpression (4, 2.5, and 8.5 times; resp.), and miR-672 down-regulation (3 fold) upon Mox-LDL treatment (see Supplementary Table III). In particular, miR-22 was upregulated 8.5 times in Mox-LDL treated cells as compared to control.

### 3.9. A Functional Relation between miR-22 Induction, HO-1 Expression, and Tubulogenesis Inhibition by Mox-LDL in HUVECs

**Figure 6:**
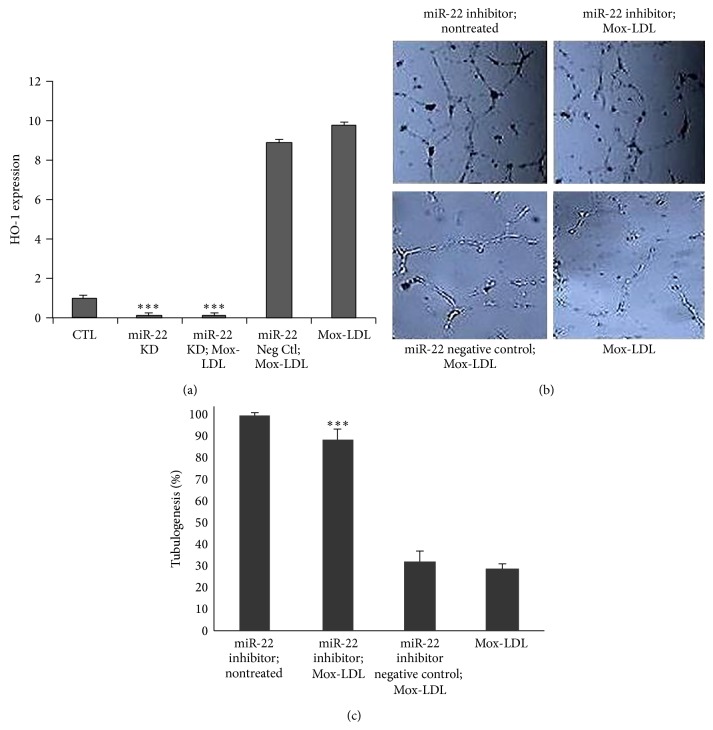
(a) Differential HO-1 expression between HUVECs submitted to different treatments: CTL (control mock-treated cells), miR-22 K.D (cells treated with miR-22 inhibitor), miR-22 K.D; Mox-LDL (cells treated with miR-22 inhibitor and Mox-LDL), miR Neg Ctl; Mox-LDL (cells treated with control miR and Mox-LDL), and Mox-LDL (cells treated with Mox-LDL). HUVECs were either treated or not with miR-22 inhibitor or miR inhibitor negative control. HUVEC were incubated for 24 hours in culture medium supplemented with mock medium (CTL) or Mox-LDL. Total RNA was extracted and analyzed by qRT-PCR using HO-1-specific primers. Pooled data from 3 independent experiments are shown. ^***^ *P* < 0.001 (Student's *t* test). (b) The effect of miR-22 knockdown on Mox-LDL treatment in HUVEC tubulogenesis. Representative fields of HUVEC cultures in tubulogenic conditions. HUVECs were either treated or not with miR-22 inhibitor or miR inhibitor negative control. Afterwards, cells were cultured in the presence or absence of Mox-LDL for 24 hours. HUVECs were then seeded on Matrigel supplemented with mock medium (CTL) or Mox-LDL (Mox-LDL) and incubated overnight. (c) Quantification of tubulogenesis in HUVEC cultures. After overnight incubation in Matrigel supplemented with mock medium (CTL) or Mox-LDL (Mox-LDL), pictures were taken of 4.906 mm square fields. Total tubule network length was then measured using the Image J computer program. Shown data are expressed as total tubule network length for each condition and normalized to the “miR-22 inhibitor; nontreated” control condition set as 100%. Mean ± SEM on 3 independent experiments performed in duplicate. ^***^ *P* < 0.001 versus Mox-LDL (ANOVA, Turkey's multiple comparison test).

As we established an effect of Mox-LDL on HUVEC motility and tubulogenesis, as well as a correlated mRNA and micoRNA signature, we assessed a causal and functional interaction between these three observations; we first screened the upregulated mRNA 3′UTR for putative consensus sites targeted by up- or downregulated miRs.* In silico* analysis uncovered a miR-22 target site located between positions 276 and 299 in the HO-1 3′UTR. In order to test a causal effect of miR-22 on HO-1 gene expression, we examined the effect of miR-22 knockdown on the expression of HO-1. As illustrated in Figure 6(a), depletion of miR-22 prevented HO-1 expression even when the cells were treated with Mox-LDL.

In order to test a causal effect of miR-22 on tubulogenesis, we prevented miR-22 expression in Mox-LDL treated ECs and assessed their tubulogenic activities in a Matrigel assay. As illustrated in Figure 6(b), inhibition of miR-22 specifically counteracted Mox-LDL negative effect on tubulogenesis. We next asked if the functionally connected miR-22 and HO-1 upregulations under Mox-LDL treatment could account for the negative phenotypical effect of Mox-LDL on tubulogenesis. We also verified that overexpression of HO-1 recapitulated the inhibitory effect of Mox-LDL treatment on angiogenic properties of HUVECs (Supplementary Figure IV).

## 4. Discussion

Our data indicate that Mox-LDL interferes negatively with HUVEC mobility (motility, migration, and tubulogenesis)* in vitro* through an increase of miR-22 and HO-1 expression.

Our study must be discussed in light of previously published studies on the effects of ox-LDL on (i) cell death/apoptosis and (ii) “angiogenesis.”

Regarding cell death, in contrast to previous reports showing that ox-LDL is able to induce apoptosis and reduce the viability of vascular ECs [29, 30], we found no effect of Mox-LDL on cell proliferation and death. This discrepancy could be related to the experimental design, cell type, and oxidation protocol or ox-LDL concentration. We used an oxidizing agent and ox-LDL concentrations that are both pathophysiological which, we believe, are more closely related to an* in vivo* situation. In particular, copper widely used to oxidize LDL* in vitro* is unlikely to be available at concentrations sufficient to generate observed ox-LDL concentrations. Previously proposed actors such as lipoxygenase or NADPH oxidase have now been discarded. Although new actors could be proposed such as VPO (peroxidasin), MPO is a favourite* in vivo* actor: it oxidizes LDL* in vitro*; high serum level is a risk factor in CVD; MPO oxidized LDL is detected* in vivo*. We used oxidized LDL concentrations that have been previously detected* in vivo*. Finally, we applied a quality control procedure assessing the* in-vivo*-like LDL oxidation levels. These conditions could elicit specific signal transduction pathways as recently illustrated by their unique ability to trigger a pathway involving PLA2 in macrophages [31].

Regarding angiogenesis, we have shown for the first time that pathophysiological concentrations of Mox-LDL induce significant functional changes in human ECs. Thus in our hands, ox-LDL inhibits HUVEC* in vitro* tubulogenesis, motility, and migration, properties associated with* in vivo* angiogenesis. This has to be confronted with the few reports regarding an ox-LDL effect on angiogenesis. These data are once again contradictory, a fact which could be again related to different experimental protocols and in particular different ox-LDL concentrations. Thus, while earlier studies showed an inhibitory effect of ox-LDL and hypercholesterolemia on angiogenesis-like endothelial growth [29], some recent studies report that ox-LDL may play a proangiogenic role at low concentrations [32, 33]. Our observations might relate to the fact that we used oxidizing agent and concentrations which are both pathophysiological to be as close as possible to* in vivo* situations.

After our phenotypical analysis, we aimed at determining the molecular mechanisms interfering with EC motility. Although the simplest hypothesis would ultimately involve an interference with adhesion foci function we could not detect any effect of Mox-LDL on EC adhesives properties.

We next analyzed changes in gene expression associated with Mox-LDL treatment. Our results suggest that the observed phenotypic effect could be explained at the molecular level by HO-1 gene expression regulation by miR-22. The HO-1 enzyme is induced in response to stress stimuli such as oxidative stress and catalyzes the degradation of heme into carbon monoxide, biliverdin, and free iron. There are numerous studies linking HO-1 to angiogenesis pathways [34–38] suggesting both positive and negative effects. Other studies also report the antiangiogenic activity of miR-22 [39–41]. Two studies in particular, one performed in a colon cancer model [42] and the other in a hepatocellular carcinoma model [43], linked respectively miR-22 and HO-1 upregulation to a decrease in cell migration, invasiveness, and wound healing, although independent of Mox-LDL treatment, but nevertheless in accordance with our observations performed with Mox-LDL treatment. Finally, several reports suggest how HO-1 could interfere with angiogenesis. It indeed induces the expression of pro- (CCL-2, CXCL-8) or anti- (CXCL-10) angiogenic chemokines. In this later case HO-1 overexpression induced secretion by EC of CXCL-10 which was able to inhibit EC growth [44]. We did not see any effect on CXCL10 expression nor on cell growth. To conclude about the role of HO-1, it is worth mentioning that none of the previous publications, although some claim that HO-1 enzymatic activity is required, investigated heme availability or CO, iron, and bilirubin production.

In order to check the causative role of the miR-22/HO-1 axis in the Mox-LDL signal transduction pathway, we inhibited miR-22 expression in ECs. MiR-22 inhibition abrogated Mox-LDL negative effect on tubulogenesis. Furthermore, miR-22 knockdown prevented HO-1 overexpression and induction by Mox-LDL. We also checked the effect of HO-1 knockdown or ectopic expression on EC angiogenic abilities. While HO-1 knockdown indicated that a minimal amount of HO-1 is mandatory for* in vitro* angiogenesis, its overexpression was deleterious. This supports previous indications of a complex role of HO-1, whose lower concentrations had stronger effects than higher concentrations [44] and indicates that appropriate levels of HO-1 are crucial for tubulogenesis.

Our data fill gaps in the Mox-LDL signal transduction pathway. Ox-LDL have been previously reported to bind to the LOX-1 scavenger receptor and to trigger signal transduction pathways involving several kinases including JNK, pKC, ERK 1 and 2, Raf, and IP3K [34, 45]. We focused on the end of this pathway: control of gene expression and phenotypic effects. Bioinformatics prediction tools pointed at a putative miR-22 target site in HO-1 mRNA and suggested a direct regulation of the latter by the first. However only rare cases of direct miR positive effects have been reported previously [46, 47]. Reporter gene assays conducted in order to assess this possibility gave indeed negative results (data not shown). Putative miR-22 target sites, detected in the HO-1 promoter, suggested a possible miR transcriptional effect as previously documented [48, 49]. Reporter gene assays did not support that suggestion either. Reports of miR positive effects through inhibition of a negative regulator are more frequent. In our case, this miR target remains to be identified. Yet, an alternative HO-1 control by miR-22 could happen through inhibition of nonsense-mediated RNA decay (NMD). For example, the brain-specific miR-128 was recently shown to induce a battery of transcripts in neuronal cells through NMD repression by targeting the exon junction complex core component MLN51 and the RNA helicase UPF1 [50].

We first rejected the 3 other miRs whose expression was affected by Mox-LDL due to the lack of target sites in the HO-1 3′UTR. In the light of our negative reporter assays, they nevertheless deserve to be considered again, as they could act indirectly (for example, by interfering with the expression of a microRNA or a protein itself acting directly on HO-1 expression). This will be addressed in our future work.

The simplest model which best explains our observation is as follows. LDL modified by MPO binds to the scavenger receptor LOX-1 on ECs. This triggers an oxidative stress signaling pathway that will stimulate the binding of NRF-like transcription factor to potential consensus sequence in miR-22 promoter [51]. This in turn increases miR-22 expression. Mir-22 indirectly increases HO-1 expression through unidentified targets possibly by repressing NMD mechanisms in the cell. This interferes with cell motility.

However uncertainties remain in this transduction pathway. First, the nature of the molecular actor linking miR-22 and HO-l is unknown. The cell specificity of this link should be addressed as we could not perform the reporter gene assays in HUVECs. Secondly, the involvement of HO-1 enzymatic activity and the nature of its target is still hypothetic.

Our data have all been obtained* in vitro*. They suggest that Mox-LDL interferes with angiogenesis* in vivo*. Thus, high LDL concentrations in patients' serum would impair angiogenesis and hinder EC ability to cope with a damaged endothelium. These mechanisms are at play in several pathological or physiological conditions. For example, high LDL levels would impair angiogenesis-dependent tumour growth, although this seems in contradiction to reports of obesity as a risk factor for some cancers. More appropriately, while it is well known that high LDL concentrations are a risk factor for cardiovascular diseases through atherosclerosis, our data suggest that they would furthermore contribute to the bad evolution of the disease through interfering with “reendothelialization” of vascular denudations, atheroma-associated wounds, stents, aneurysms, infarcts, or places of restenosis. More generally high ox-LDL levels could also negatively impact the progression of all the pathologies associated with endothelial desquamation, such as vasculitis, lupus erythematosus, sickle cell anemia, thalassemia, septic shock, infections (rickettsiae, CMV), or cancer.

## 5. Conclusion

Atherosclerosis chiefly contributes to cardiovascular disease, the first cause of death worldwide, which includes cerebral vascular accidents and myocardial infarctions. Atherosclerosis is linked to the accumulation of oxidized LDL in the vascular wall, affecting its function. We show that pathophysiological concentrations of oxidized LDL impair the mobility and the angiogenic capability of endothelial cells. We identify a microRNA (a small RNA able to tune gene expression), miR-22, and the gene coding for heme oxygenase 1 whose overexpression is instrumental in this phenotypical effect. Our results suggest that lipid rich diets that enhance the LDL and ox-LDL levels not only cause atheroma plaque formation but also interfere with the endothelial wound healing process; they can therefore contribute to the progression of the disease; they can influence negatively all clinical conditions associated with endothelial desquamation. They can interfere with denudations resulting from medical protocols such as catheterization or stenting.

## Supplementary Material

We showed that Mox-LDL inhibits EC motility and tubulogenesis and tried to uncover the molecular mechanisms behind this action. Supplementary Table I quantifies the expression changes of 94 genes known to be involved in angiogenesis under this Mox-LDL treatment while supplementary Tables II and III summarize the changes detected in miR expression. We showed in the *«*results*»* section that two of these expression changes are important for Mox­LDL action, namely those of HO-I and miR-22. For this purpose we used miR-22 inhibitors and supplementary Figure III illustrates a control experiment that showed that it indeed reduced Significantly miR-22 levels. We also overexpressed HO-I and showed that this was sufficient to mimick the action of Mox-LDL (supplementary Figure IV). We also attempted to uncover the final targets of HO-I action, and supplementary figures I and II suggest that this act ion is not mediated by a change in the expression or localization of the adhesion plaque components.

## Nonstandard Abbreviations and Acronyms

LDL: Low density lipoprotein
MPO: Myeloperoxidase
ox-LDL: Oxidized low density lipoprotein
Mox-LDL: Myeloperoxidase oxidized low density lipoprotein
HO-1: Heme oxygenase 1
EC: Endothelial cell.
