# Supplementary material for: Myeloperoxidase Oxidized LDL Interferes with Endothelial Cell Motility through miR-22 and Heme Oxygenase 1 Induction: Possible Involvement in Reendothelialization of Vascular Injuries
Source: Mediators Inflamm. 2014 Nov 2;2014:134635. doi: 10.1155/2014/134635 (PMC4233670; doi:10.1155/2014/134635)
Supplement: Supplementary file 1 — We showed that Mox-LDL inhibits EC motility and tubulogenesis and tried to uncover the molecular mechanisms behind this action. Supplementary Table I quantifies the expression changes of 94 genes known to be involved in angiogenesis under this Mox-LDL treatment while supplementary Tables II and III summarize the changes detected in miR expression. We showed in the «results» section that two of these expression changes are important for Mox­LDL action, namely those of HO-I and miR-22. For this purpose we used miR-22 inhibitors and supplementary Figure III illustrates a control experiment that showed that it indeed reduced Significantly miR-22 levels. We also overexpressed HO-I and showed that this was sufficient to mimick the action of Mox-LDL (supplementary Figure IV). We also attempted to uncover the final targets of HO-I action, and supplementary figures I and II suggest that this act ion is not mediated by a change in the expression or localization of the adhesion plaque components. [file 134635.f1.pdf]

Supporting information

Supplementary figures

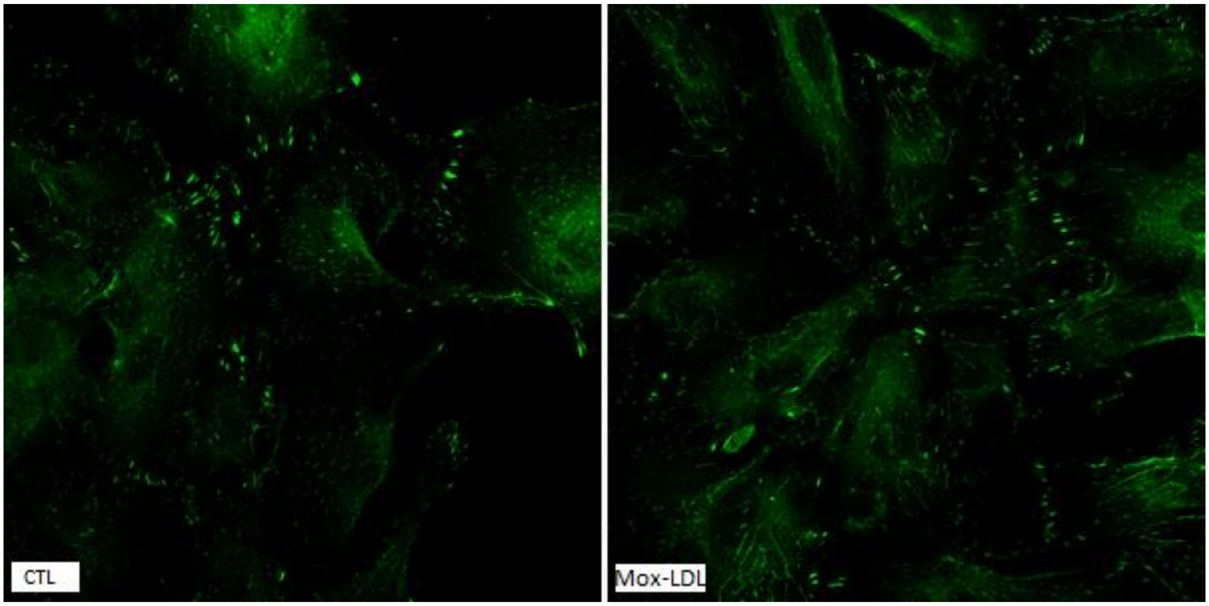

Supplementary Figure I. Control (CTL) mock-treated and Mox-LDL (Mox-LDL) treated HUVECs were subjected to confocal microscopy analysis to detect the patterning of focal adhesions using paxillin IF staining. Cells were cultured in the presence or absence of Mox-LDL for 24 hours, fixed and processed for immunofluorescence.

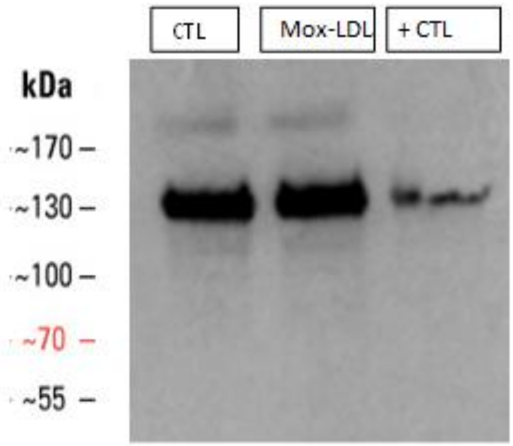

Supplementary Figure II. Western blot analysis of control (CTL) mock-treated and Mox-LDL (Mox-LDL) treated HUVECs using an anti phospho-FAK antibody. Cells were cultured in the presence or absence of Mox-LDL for 24 hours. HepG2 protein extract was used as a positive control (+ CTL).

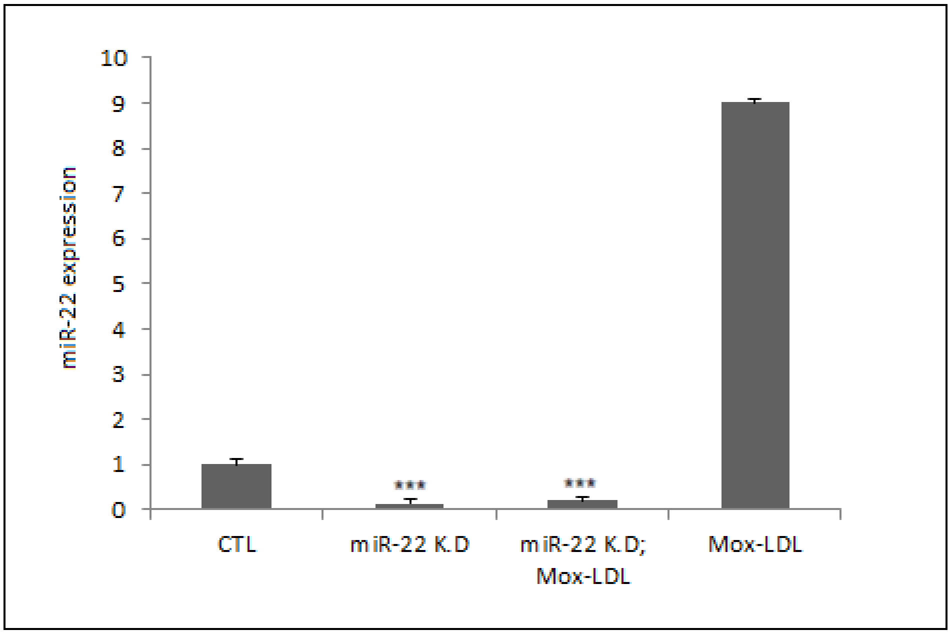

Supplementary Figure III. Differential miR-22 expression between HUVECs submitted to different treatments: CTL (control mock-treated cells), miR-22 K.D (cells treated with miR-22 inhibitor), miR-22 K.D ; Mox-LDL (cells treated with miR-22 inhibitor and Mox-LDL) and Mox-LDL (cells treated with Mox-LDL). Total RNA was extracted and analyzed by qRT-PCR using miR-22-specific primers. Pooled data from 3 independent experiments are shown. ***, $p < 0.001$ (Student's *t* test).

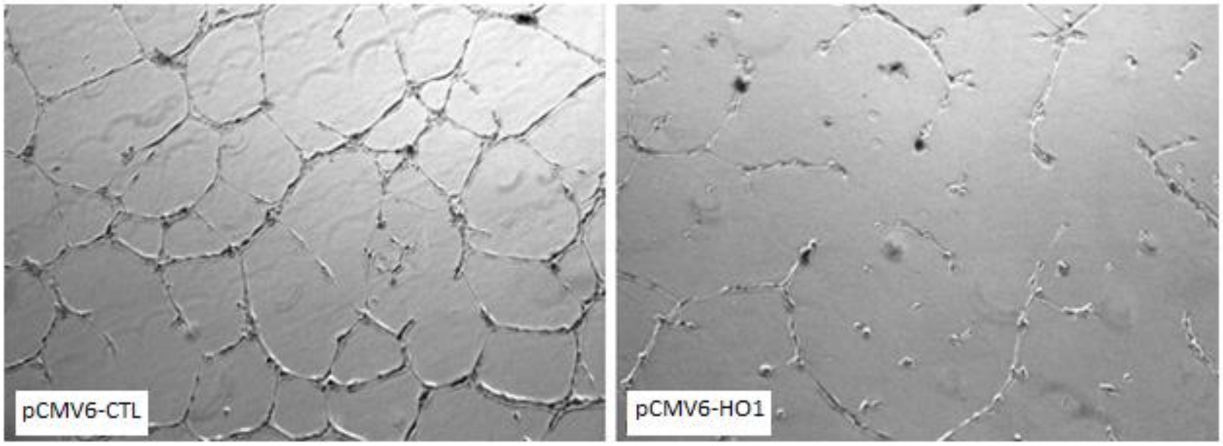

Supplementary Figure VI. The effect of HO-1 overexpression on HUVEC tubulogenesis . A) Representative fields of HUVEC cultures in tubulogenic conditions. HUVECs were transfected with either a control empty pCMV6 vector or the same vector containing HO-1 ORF for overexpression. Afterwards, cells were cultured for 24 hours. HUVECs were then seeded on MATRIGEL™ and incubated overnight.

Supplementary tables

Rank	Gene Name	Fold Change
1	HMOX1 (HO-1)	11.098827
2	ANGPTL4	3.54599
3	FLT1	-2.048766
4	TNF	49.373841
5	ANG	1.862989
6	ERBB2	2.042583
7	GDF5	-2.042658
8	ITGB3	-2.446958
9	F3	2.259589
10	MMP14	1.94062
11	STC1	-4.21923
12	VEGFC	-1.617768
13	MET	-1.926857
14	PTEN	-1.994231
15	CXCR4	1.463703
16	BIRC5	-1.896081
17	IL18	1.734897
18	PTN	7.617571
19	ENG	2.058152
20	AKT1	1.793787
21	CTGF	-1.834454
22	FGF2	-1.461817
23	ITGB1	-1.794913
24	IL6	-1.533378
25	ITGAV	-1.44376
26	HIF1A	-1.273682
27	MYC	-2.207365
28	TNFSF15	1.521195
29	HOXD3	1.334072
30	CSF1	1.704672
31	KDR	-1.601133
32	EPHB4	1.568042
33	CAV1	-1.571543
34	Hs18s	1.338716

35	FLT4	1.591639
36	VEGFA	-1.219629
37	BMX	-1.529389
38	IGFBP3	-1.44343
39	ANGPT1	-2.116423
40	ANPEP	1.195922
41	TEK	-1.472871
42	EFNB2	-1.356693
43	FIGF	1.223317
44	PECAM1	-1.329769
45	PGF	1.71445
46	AGTR1	1.815252
47	RAC1	-1.151049
48	HSGenomic	1.767624
49	SPP1	2.69479
50	DLL4	-1.151107
51	ANGPT4	-2.084858
52	ACVRL1	1.620393
53	NOS3	-1.208613
54	VEGFB	1.116112
55	ANGPT2	-1.166612
56	EPHA2	1.337519
57	EDN1	-1.148181
58	ETS1	-1.376352
59	COL18A1	1.180191
60	ANGPTL6	1.120029
61	SSTR2	-273.450392
62	HPSE	-1.070885
63	PDGFB	1.621356
64	TGFB1	-2.243904
65	TIMP1	-1.176056
66	CEACAM1	-1.058709
67	HGF	1.853062
68	MIF	1.281158
69	SPARC	1.040428
70	SERPINE1	1.250923
71	NOS2	-215.498894

72	PTGS2	1.064404
73	NRP1	1.018726
74	ITGA5	1.141821
75	THBS1	1.067319
76	CYR61	-1.12108
77	MAPK7	1.153725
78	EPOR	1.097062
79	TYMP	1.156345
80	STAT3	1.097638
81	VASH1	1.088242
82	TP53	-1.02341
83	ID1	1.030433
84	BSG	1.117271
85	MMP2	1.046882
86	CXCL12	1.366611
87	SERPINF1	-1.06559
88	ANGPTL3	-1.403216
89	CCL2	1.01938
90	MMP9	-1.0471
91	IL1B	2.809877
92	ANGPTL1	1.954176
93	FGF1	1.520392
94	SERPINB5	15.988917
95	CXCR2	8.999235
96	LEP	-261.630054

Supplementary Table I. Shows the results of the human Angiogenesis StellARray™ technique which was used to study the expression of 94 pre-selected genes involved in angiogenesis pathways. The table shows the complete list of genes with their fold change in expression levels in Mox-LDL treated condition as compared to the control condition (control mock-treated). Treatment of HUVECs with Mox-LDL significantly altered the expression of only one gene, the Heme Oxygenase 1 (HO-1) gene, which saw an 11-fold increase in its expression level.

**microRNA**	**Fold Change**
hsa-miR-107	2.4313
hsa- miR-133a	6.935
hsa- miR-134	0.3036
hsa- miR-188-3p	3.3762
hsa- miR-200b	3.9903
hsa- miR-203	16.5497
hsa- miR-22	550.8621
hsa- miR-29c	2.124
hsa- miR-449a	0.5709
hsa- miR-449b	0.5709
hsa- miR-502-3p	11.3658
hsa- miR-505	0.0081
hsa- miR-518b	0.0947
hsa- miR-629	8.2102
hsa- miR-636	0.1267
hsa- miR-652	0.2063
hsa- miR-672	0.19
hsa- miR-708	3.7797

Supplementary Table II. TLDA results showing the list of miRs that were identified to be differentially expressed exclusively in Mox-LDL treated HUVECs with their fold change in expression levels as compared to the control condition (control mock-treated). HUVEC were incubated for 24 hours in culture medium supplemented with mock medium, native LDL or Mox-LDL. Total RNA was extracted and analyzed by making use of the Taqman Low Density Array (TLDA) method.

microRNA	Fold Change
hsa- miR-133a	4.021
hsa- miR-203	2.480
hsa- miR-22	8.503
hsa- miR-672	0.331

Supplementary Table III. Shows the validated list of miRs that were confirmed to be differentially expressed exclusively in Mox-LDL treated HUVECs with their fold change in expression levels as compared to the control condition (Control mock-treated). HUVEC were incubated for 24 hours in culture medium supplemented with mock medium, native LDL or Mox-LDL. Individual qRT-PCR was used to validate the results of the TLDA high-throughput experiments. Total RNA was extracted and analyzed by qRT-PCR conducted on each of the differentially expressed miRs using specific primers.
